# Automatically exposing OpenLifeData via SADI semantic Web Services

**DOI:** 10.1186/2041-1480-5-46

**Published:** 2014-11-19

**Authors:** Alejandro Rodríguez González, Alison Callahan, José Cruz-Toledo, Adrian Garcia, Mikel Egaña Aranguren, Michel Dumontier, Mark D Wilkinson

**Affiliations:** Centro de Biotecnología y Genómica de Plantas, Universidad Politécnica de Madrid, Madrid, Spain; Center for Biomedical Informatics Research, Stanford University, Stanford, CA USA; Department of Biology, Carleton University, Ottawa, ON Canada; Genomic Resources Group, University of the Basque Country (UPV-EHU), Bilbao, Spain

**Keywords:** OpenLifeData, Bio2RDF, SADI, Semantic web services, SPARQL, SHARE, Sentient knowledge explorer, Galaxy

## Abstract

**Background:**

Two distinct trends are emerging with respect to how data is shared, collected, and analyzed within the bioinformatics community. First, Linked Data, exposed as SPARQL endpoints, promises to make data easier to collect and integrate by moving towards the harmonization of data syntax, descriptive vocabularies, and identifiers, as well as providing a standardized mechanism for data access. Second, Web Services, often linked together into workflows, normalize data access and create transparent, reproducible scientific methodologies that can, in principle, be re-used and customized to suit new scientific questions. Constructing queries that traverse semantically-rich Linked Data requires substantial expertise, yet traditional RESTful or SOAP Web Services cannot adequately describe the content of a SPARQL endpoint. We propose that content-driven Semantic Web Services can enable facile discovery of Linked Data, independent of their location.

**Results:**

We use a well-curated Linked Dataset - OpenLifeData - and utilize its descriptive metadata to automatically configure a series of more than 22,000 Semantic Web Services that expose all of its content via the SADI set of design principles. The OpenLifeData SADI services are discoverable via queries to the SHARE registry and easy to integrate into new or existing bioinformatics workflows and analytical pipelines. We demonstrate the utility of this system through comparison of Web Service-mediated data access with traditional SPARQL, and note that this approach not only simplifies data retrieval, but simultaneously provides protection against resource-intensive queries.

**Conclusions:**

We show, through a variety of different clients and examples of varying complexity, that data from the myriad OpenLifeData can be recovered without any need for prior-knowledge of the content or structure of the SPARQL endpoints. We also demonstrate that, via clients such as SHARE, the complexity of federated SPARQL queries is dramatically reduced.

## Background

Data integration is an ongoing challenge for biological informaticians, and is often a study unto itself, with numerous research groups worldwide approaching the problem from a variety of perspectives [1]. Integration is difficult for a variety of reasons, generally broken into the three core issues of syntax, structure, and semantics [2]. In addition, assigning and using unique identifiers for data items and concepts is an essential requirement in biology and elsewhere, and forms an equally disruptive barrier to successful integration [3]. Syntactic barriers include issues such as binary or textual format, and free-text or structured text; structural barriers involve such things as flat-file formats, and XML Schema; semantic barriers include inconsistent naming, naming conflicts (multiple things with the same name, or multiple names for the same thing) or insufficiently defined names; and finally identification issues involve non-unique identifiers, identifiers that can only be interpreted within a particular scope (e.g. in the context of a given database), non-opaque identifiers, and unstable or unpredictable identifiers.

The Semantic Web Initiative [4] has recently emerged with technologies and frameworks aimed at solving at least some of these problems. In particular, the Resource Description Framework (RDF [5]) is an entity-relationship data model that is, in principle, machine-readable and capable of representing any concept or data entity. RDF also proposes several approved syntaxes, aimed at maximizing machine-readability. Importantly, a query language has been developed for RDF - the SPARQL Protocol and RDF Query Language (SPARQL [6]) - and a protocol for exploring and retrieving the RDF stored in “SPARQL endpoints” on the Web is now well-established and, in our experience, highly consistent from implementation to implementation.

With RDF as its core, the Linked Data initiative [7] proposes several best practices that dramatically improve the discoverability and integration of data on the Web. First, all data entities and relationships must be identified by a Uniform Resource Identifier (URI), which guarantees uniqueness on a global scale. Second, URIs should resolve to data and metadata using the most common Web protocol, HTTP. Third, URIs should resolve to useful/informative information, and resource providers should offer this information in a variety of syntaxes that can be selected by HTTP content-negotiation; in particular, Linked Data resources should provide a means of retrieving the data in the form of RDF. Finally, this RDF should contain labeled links between that piece of data, and other pieces of data also identified by resolvable URIs, where the links indicate the relationship between the two data elements and are, themselves, resolvable URIs. With the ability to retrieve, share, and re-use these relationship definitions, we begin to move towards the “semantic” aspect of the Semantic Web.

Attempts to unify semantics have long been a focus of biomedicine. The medical world has engaged for centuries in the development of nosologies for naming and classifying diseases. Within the bioinformatics community, ontologies have become widely adopted in the past decade, with the most prominent of these being the Gene Ontology [8]. While such ontologies generally focus on consistent and sensible human-readable names, they have dedicated less attention to the unique identification of the concept - names in ontologies are not guaranteed to be globally unique, nor are concepts guaranteed to be uniquely named, and can appear in multiple ontologies. However, as these ontologies became encoded using the rules of Linked Data, aspects of these problems were also solved. Concepts became globally and uniquely identified by resolvable URIs, and shared concepts could be referred to by URI from one ontology to another. Moreover, modern ontologies’ use of the Web Ontology Language (OWL) [9] description logic to define the meaning of the “links” in Linked Data - effectively, the precise nature of the relationship between one data entity and another - enabled machines to automatically traverse these linkages in a meaningful way.

Two key issues remained problematic, however, even with Linked Data. First there was no widely-used mechanism to ensure the stability and predictability of URIs representing data and concepts - for example there was no way to predict the URI for the Protein Data Bank record of the Arabidopsis UFO protein, and even if this URI were determined, it might not be the same from one day to the next. As a result, individual Linked Data resources could not reliably link out to other Linked Data resources, because the URIs were unpredictable and unstable. Data tended to remain “siloed” even in the Linked Data world because links generally pointed inward, rather than outward, as a result of this instability and unpredictability. Second, the structure of what was returned when the URI to a piece of data was resolved was also not sufficiently predictable, and not consistent from site to site, even for the same type of data. While Linked Data is a significant improvement over XML Schema with respect to the predictability of its data structures, there were still no guidelines for how to arrange the relationships between pieces of data, or even what those relationships could/should be. It was these remaining problems that became the focus of the Bio2RDF project.

Bio2RDF is an open source project that uses Semantic Web technologies to create a sustainable infrastructure for publishing biological data in a manner that eases the task of data integration [10–12]. Bio2RDF scripts convert heterogeneously formatted data (e.g. flat-files, tab-delimited files, dataset specific formats, SQL, XML etc.) into RDF. Bio2RDF follows a set of basic conventions to generate and provide Linked Data which are guided by Tim Berners-Lee’s design principles and a set of community-established guidelines and practices. Specifically, entities, their attributes and relationships are named using a simple convention to produce Internationalized Resource Identifiers (IRIs) that are highly predictable in their structure, while statements are articulated using the lightweight semantics of RDF Schema (RDFS) and Dublin Core. Bio2RDF, however, did not reliably implement all of the requirements of “well behaved” linked data, such as HTTP content-negotiation, and had somewhat limited expressivity in its relationships as a result of using the semantics of RDF Schema. OpenLifeData provides customized services over Bio2RDF SPARQL endpoints. Its goal is to provide alternative user interfaces and application programming interfaces to Linked Open Data beyond what Bio2RDF currently does. OpenLifeData enriches Bio2RDF’s RDFS semantics to OWL expressivity, implements rich HTTP content-negotiation, and utilizes query-rewriting to resolve OpenLifeData IRIs and SPARQL queries against the Bio2RDF SPARQL endpoints.

OpenLifeData data is accessed by users either via the Web, through resolution of a URI to an HTML-representation of its data content in their browser, or by the submission of a SPARQL query to one of the OpenLifeData endpoints. While the data-types and relationships within each endpoint can be determined by manual exploration of the endpoint, SPARQL queries must nevertheless be constructed manually, and then posed against the appropriate endpoint(s). Extracting OpenLifeData Linked Data, therefore, remains a non-trivial task for even experienced bioinformaticians.

The 2014 release of OpenLifeData (based on Release 3 of Bio2RDF) developed a scheme to provide a pre-computed summary, or index, of the contents of each OpenLifeData SPARQL endpoint in order to reduce the computational load required for exploratory queries and enable new applications. Summary metrics were pre-computed, including number of triples, number of unique subjects, number of unique predicates, number of unique objects, list and frequency of unique types, list and frequency of unique predicates, list and frequency of unique subject, predicate-unique object tuples, list and frequency of instances of subject type, predicate, and instances of unique object type, and finally number of links to other datasets. These indexes make it easier to determine the structure and content of each OpenLifeData endpoint, and moreover, the structures are highly consistent from endpoint to endpoint.

Semantic Automated Discovery and Integration (SADI) [13] is a set of design principles for exposing Web Services in a manner that simplifies their integration with other Semantic Web resources. Described simply, SADI Services are Web-based tools that consume a particular type of data, and return another type of data that is explicitly related to that input. For example, you could send DNA sequences to a SADI tblastx service, and it would give you back Protein sequences that are connected to the original DNA sequence by the “hasProteinHomologyTo” relationship. Expressed more concretely, SADI services consume and produce RDF data, where instances of an input OWL class, represented in RDF, are submitted to the service by HTTP POST, and RDF instances of an output OWL class are returned in response. The constraint SADI places on these data is that the output class must be a specialization of the input class such that the input instances are related to the new service-generated data nodes through ontologically-defined relations. The result of chaining SADI services together, therefore, is an unbroken network of well-formed and ontologically-grounded Linked Data, which can be explored and traversed using standard tools such as SPARQL.

SHARE (Semantic Health And Research Environment) [14, 15] is a SADI client that combines: a registry of the input and output OWL classes for all known SADI services, a service discovery and invocation API, an automated workflow design and enactment engine, and a logical reasoner. While other components of SHARE are discussed in detail in the previously-referenced papers, it is relevant to this manuscript that service discovery is achieved by indexing all known SADI services in the SHARE registry, such that input types, output types, and the properties that link them, are all rapidly searchable. This registry is made publicly available as a SPARQL endpoint, where the data model of the registry follows that of the myGrid serviceDescription [16] ontological class.

The similarity between the input-type, property, output-type “signature” of a SADI Web Service, and the subject-type, predicate, object-type indexes of the OpenLifeData endpoints provides a natural mechanism through which these two initiatives could be combined, such that OpenLifeData becomes discoverable and accessible via SADI. At the NBDC/DBCLS BioHackathon 2013 we proposed that it should be possible to automatically generate (a) formalized definitions of SADI services, (b) SPARQL queries to retrieve the service-appropriate data from the OpenLifeData endpoints, and (c) the SADI service code to serve that data, all by simply parsing the OpenLifeData indexes. This manuscript describes the realization of that vision. For the remainder of the manuscript will use the short name ‘OpenLifeData2SADI’ as a convenient way of referring to the project as a whole.

## Implementation

OpenLifeData’s content summaries are provided as RDF [17]. We utilize the Jena [18] Java libraries to parse the [Subject type - predicate - Object type] (SPO) triple patterns in these indexes, and additional indexes created specifically for this project, to generate sets of three configuration files used by OpenLifeData2SADI to serve each data-type within OpenLifeData. The first file contains two OWL ontological classes, describing the input and output data for the service. These ontologies are published on the Web such that the input and output class URIs are resolvable through HTTP GET. The second file is a summary containing the URIs of the input and output OWL Classes for that service, the human-readable class names, and the URI and name of the RDF predicate that links the two classes. Finally, a third file is generated that contains a SPARQL query template that, when filled-in with data and executed against the appropriate OpenLifeData endpoint, retrieves the output data appropriate for that service. We now describe in additional detail how each of these steps is undertaken.

## Parsing the indexes

Each OpenLifeData dataset is served from its own SPARQL endpoint, and contains data within a specific namespace (e.g. ‘sgd’ for Sacharromyces Genome Database, or ‘ncbigene’ for the NCBI Gene). The content of each endpoint has been pre-indexed, using VoID (Vocabulary of Interlinked Datasets), where the index captures all unique data-type/predicate/data-type triples for that endpoint. For example, one of the index triples for the HGNC endpoint is “Gene Symbol/x-omim/Gene”. The Java collector first parses the information provided in the OpenLifeData indexes to obtain two parameters: SPARQL Endpoint URL and Namespace - effectively, the location of each dataset, and the domain/scope of that dataset. In principle, each endpoint could be interrogated to retrieve all SPO patterns by executing the following SPARQL query:


This would be sufficient to gather all information necessary to create SADI services that output resource nodes (URIs); however, at this time, OpenLifeData does not index the large component of data that exists as literal values (numbers and strings). As such, to be fully comprehensive, we execute an iterative set of queries over each endpoint which gathers all subject-types, then the predicates associated with each subject-type, and finally the object type that is connected by each predicate, including the cases where the object is a literal value. To further enrich the semantics, we then do federated queries over multiple end-points in an attempt to determine more specific details about the object types. For example, the omim dataset includes links to entities in the hgnc dataset, but considers all of these to be “Resources” - a generic term for something that exists in another dataset. Through our federated queries, we can determine that these hgnc “Resources” represent, for example, Genes, or SNPs, and thereby we are able to construct semantically richer descriptions of what the SADI services will consume/produce.

The queries we execute (in template form) are as follows:

Get Subject-types


Get Predicate-types


Get Object-types


Get Data-types


Federated Query for object types


## Configuration file creation

After retrieving all SPO patterns for each endpoint, OpenLifeData2SADI then builds the files needed to automatically configure the SADI Service; each SPO triple pattern becomes its own Service, where the service consumes data of the ‘Subject’ type, and returns all triples from that endpoint matching the SPO pattern for that Subject. For each Service, three configuration files must be created:

### Input and output ontology classes

Using the Java OWL API [19] we create ontology classes based on the pattern of each SPO in each endpoint; these classes describe the OWL properties required for/provided by the Input and Output of the service respectively.

In OpenLifeData URIs the class/predicate identifier, and namespace are separated either by the hash (#) or colon (:) characters. Since we intend that OpenLifeData2SADI services should “make sense” to both machines and humans, an attempt is made to construct a human-readable name for each class and property. The code first attempts to resolve the URI to retrieve its rdf:label, and this label, if available, is used as the human readable class/property name in the final configuration file for that service. If no label can be retrieved, the hash or colon separator is used to split a name from the rest of the URI and this is used as the human-readable name. While not entirely successful, this is our best attempt at automatically building services that have accurate human-readable descriptions.

The input class (generically called ‘Subject_Class’ in this discussion) is defined in OWL, simply, as the rdf:type of the Subject of the SPO triple, as defined by OpenLifeData. It contains no other axioms or restrictions. The class representing the output of the service is then defined as the Subject_Class with an additional property defined by the Predicate of the SPO triple, where the range of that predicate is defined by the Object data-type component of the SPO triple. This is represented in Manchester Syntax as follows:


Logically, therefore, the Service output is a subclass of the Service input (Subject_Class), as is typical for all SADI services. A similar approach is taken for OpenLifeData predicates with Literal value ranges. The resulting ontology is then saved to the local filestore with the naming convention ./<namespace>/<subject_predicate_object > .owl and this is published on the Web such that the URIs in that ontology resolve correctly.

In a second phase, the process above is duplicated, but in this second iteration, the owl:Inverse of the Predicate is used, and Subject and Object are reversed. This allows us to automatically create SADI services that traverse the OpenLifeData in either orientation, and thus behave in a manner akin to conventional SPARQL, where either Subject or Object may be bound in a constraint clause of the query.

### Configuration file

This file contains parameters required to properly configure the SADI service such that it (a) serves the appropriate data using the appropriate descriptors, and (b) provides its own metadata in a form that is comprehensible to humans. The Java code that creates these configuration files requires a single argument - the root URL to the final location of the ontologies (created above) on the Web. The configuration file contains the following parameters:

- INPUTCLASS_NAME: The name of the input class after removing the namespace. In cases where the class name is opaque or numerical, an attempt is made to resolve the class URI to its full OWL-RDF definition, and retrieve the “label” property, such that the class name is human-readable.
- INPUTCLASS_URI: The URI of the input class
- OUTPUTCLASS_NAME: The name of the output class. This is the same for all services, but conflicts are avoided since each output class name exists in a unique namespace (ontology); every output class is named “#ServiceOutput”.
- OUTPUTCLASS_URI: The Web-resolvable URI of the output class. This URI is generated by the concatenation of the root URL, the namespace of the OpenLifeData dataset, the path and name of the ontology file, and the generic class-name “#ServiceOutput”.
- PREDICATE_NAME: The name of the predicate. As with the input class name, an attempt is made to retrieve the human-readable label of the predicate if it appears that the predicate is somehow opaque or numerical.
- PREDICATE_URI: The URI of the predicate.
- ORIGINAL_ENDPOINT: The URL of the original endpoint indexed by OpenLifeData.
- GENERIC_ENDPOINT: The endpoint that should be queried by the SADI service using SPARQL. OpenLifeData is duplicated in several locations; the preferred location to query would be the value of this field.
- OUTPUT_CLASS: The rdf:type of the data that will be added during the service execution.

The resulting file is written to the local filestore in the same folder as the ontology file, with the naming convention./<namespace>/<subject_predicate_object > .cfg.

## SPARQL query file

The third file generated by OpenLifeData2SADI contains the SPARQL query that should be executed within the business logic of the SADI Web service. The content of this query is service specific, but follows the pattern:


where < PREDICATE_NAMESPACE > is replaced with the namespace of the predicate provided by the SPO, and < predicate > element is replaced with the local name of the predicate (the component after the ‘#’ or ‘:’ character). %VAR is left in the query template, and will be substituted by the SADI service at run-time, based on the input data.

In the case of the SPARQL query, there is no difference between the ‘forward’ Predicate and the inverse predicate. Inverse predicates do not exist in the OpenLifeData SPARQL endpoints, but rather are simply defined in the OWL logic that defines the entities and relationships in those endpoints. As such, we rely on logical reasoning to determine that an inverse invocation can be solved equally well by a ‘forward’ query; thus the query that serves both forward and inverse services is identical.

## SADI service implementation

To serve the OpenLifeData data, a single Perl script using the standard SADI::Simple code libraries act as the SADI Service Daemon for all services. The script listens for HTTP calls to URLs of the form:


In this URL, SADI is the name of the OpenLifeData2SADI Service script, while the additional path information (namespace and service name) are used as keys to access the configuration file and SPARQL query file appropriate for that service, as described above. The SADI Perl script parses these files, and configures itself to be capable of:

HTTP GET:

- Returning the complete service interface definition, represented as an owl:Individual of the mygrid ontology ServiceDescription Class, as per the SADI design patterns.

HTTP POST:

- Parsing the input data, which arrives in RDF syntax as owl:Individuals of that service’s Input OWL Class.
- Executing the SPARQL query, extracted from the configuration files, against the correct OpenLifeData endpoint for that service, using each of the incoming owl:Individuals to fill the query variables for that particular invocation.
- Constructing owl:Individuals compliant with the class definition of that service’s Output OWL Class, and passing this data back to the caller.

This is all accomplished using the normal SADI service template [13]. The key difference is that the Service’s interface template retrieves its values from a dynamic look-up of data from the configuration files, rather than being hard-coded into the service.

## Service registration

Two scripts were written to automate the registration and deregistration of the full suite of OpenLifeData2SADI. The registration code and deregistration code are available in the Perl folder of the GitHub project (see “Availability and Requirements” section). They operate by querying all of the configuration files (for registration) or all of the existing SHARE registry entries (for deregistration) and triggering the registry to call GET on each service endpoint. The registry functions by creating a service if it finds a valid service description document at that endpoint, or deregistering a service if it does not. Therefore, in the case of registration, the SADI script should be installed on the designated service endpoint first, in order to respond to the registry calls. In the case of deregistration, the SADI Service code should be removed prior to running the deregistration script.

## Workflows of Bio2RDF services

The establishment of the OpenLifeData2SADI suite of services made it possible to more easily explore the interconnections between OpenLifeData endpoints. In order to generate an exhaustive list of these connections, to assist third-parties in building novel exploration tools, the following query was issued which creates a list of all valid service-output to service-input pairs within the set of OpenLifeData services (note that the PREFIX directives in this example are shared for all queries in this manuscript, and will not be repeated in later examples):


Since this, in principle, represents the complete set of potential workflow connections that could be constructed within these services, we chose to formally represent the output of this query as an abstract workflow template, using the Open Provenance Model for Workflows (OPMW) Abstract Template ontology [20, 21]. Those interested in generating a copy of this abstract template for their own exploration can simply execute the OpenLifeData2SADI2OPMW.pl script in the GitHub project, which will generate a copy based on the contents of the public SHARE registry. A copy generated at the time of writing is also available in the project’s Git repository (see “Availability” section).

## Provenance

Provenance of data is becoming increasingly important as datasets get larger, more dispersed over the Web, and as data gathering and analyses become more automated. The OpenLifeData2SADI project has selected the NanoPublication [22] conventions and model for passing provenance information to the client, along with the results of their service invocation. As with all SADI services, this is achieved through normal HTTP content negotiation. If the client passes an “Accept: application/n-quads” HTTP header, the OpenLifeData2SADI service will respond by returning three named graphs, constructed according to the NanoPublication specifications. One graph contains the service output, the second contains the metadata describing the service and, for example, its name, description, and URL, and the third describing the date and time the NanoPublication was generated.

## Results and discussion

At this time there are more than 22,000 OpenLifeData2SADI services from 26 independent endpoints, and more will be generated as OpenLifeData expands into new data-types. These services are discoverable through simple queries against the SHARE registry, or through a variety of client applications. We now demonstrate the utility of the OpenLifeData2SADI application by a series of walkthroughs, where the process of discovery, execution, and chaining-together of SADI-wrapped OpenLifeData services is described in more detail and compared to the interrogation of OpenLifeData directly via SPARQL.

We will start with a small fragment of RDF data representing a Human Gene Naming Committee (HGNC) Gene Symbol:


### Discovery of OpenLifeData2SADI services

Discovery of services is generally accomplished by executing a SPARQL query against the SHARE registry [23]. Discovery of the OpenLifeData2SADI services can be accomplished by a wide variety of query structures, but in this example we will query for services that consume OpenLifeData HGNC Gene Symbols and have “approved-name” somewhere in the service’s descriptive text. The query is:


This returns a single result, which is the URL for service “hgnc_vocabulary_Gene-Symbol_hgnc_vocabulary_approved-name_string”.

### Invocation of OpenLifeData2SADI services

Invocation of a discovered OpenLifeData data retrieval service simply consists of sending the data to the service endpoint using HTTP POST. This can be accomplished with widely available tools such as Unix ‘curl’. Below, the sample HGNC Gene Symbol record described earlier, is in the file sampledata_hgnc.rdf. Curl is then used to invoke the service, as follows:


the result of this service invocation is the output data, containing the approved name from the OpenLifeData HGNC endpoint:


### Client applications

We do not expect that our users will typically discover or access OpenLifeData2SADI services via SPARQL queries or the command-line. More commonly, the same discovery and invocation interactions presented in their raw form above are presented to the user graphically via one of the SADI plug-ins or client applications; nevertheless, discovery and invocation happens the same way as described above, regardless of the client. We believe that this simple standardization provides a very low barrier-to-adoption for new users and tool-developers who wish to gain access to the myriad OpenLifeData resources.

There are a wide range of graphical clients capable of executing SHARE registry queries in response to the user’s contextual needs, or in some cases, fully automatically. We will now present several of these applications, showing how OpenLifeData services can be accessed and chained-together within these diverse clients.

**Figure 1:**
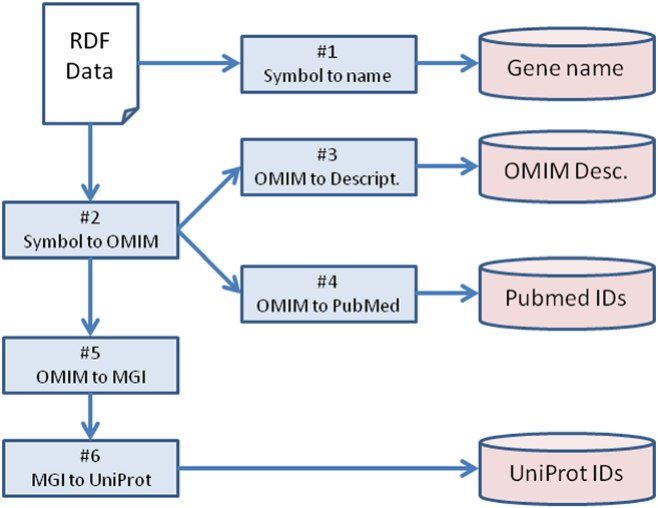
**A workflow of OpenLifeData2SADI services,** **numbered as in the list of services above, and the output data that will result.**

The list of services we will use for this demonstration are:

1. Gene-Symbol_approved-name
2. Gene-Symbol_x-omim
3. Gene_gene-function
4. Gene_article
5. Gene_x-mgi
6. Gene_x-uniprotServices (1) and (2) link an HGNC resource to its approved name and a linked OMIM entry, services (3), (4), and (5) link an OMIM resource to a gene function description, its associated PubMed entries, and its associated Mouse Genome Informatics (MGI) Gene, while service (6) links an MGI gene identifier to its associated UniProt identifier. The template workflow connecting these services in a biologically-meaningful way is shown in Figure 1.

### IO informatics knowledge explorer

**Figure 2:**
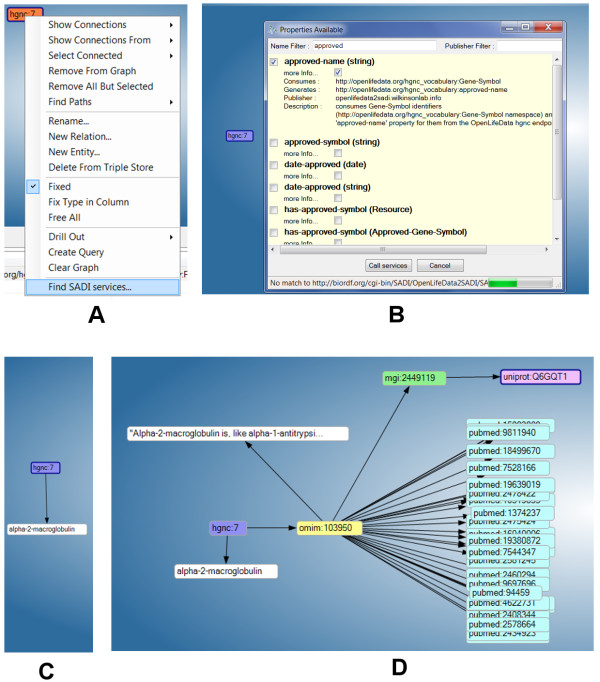
**Discovery and invocation of OpenLifeData2SADI services using the SADI plugin to the Sentient Knowledge Explorer. A**. Data nodes respond to a right-click with a context menu item “Find SADI Services”. **B**. a set of services capable of consuming nodes of that type are discovered and presented in a menu-like manner. **C**. the result of selecting the “approved-name” service from the menu. **D**. the output after iteratively invoking all 6 of the services from the example service list (effectively, manually executing the workflow in Figure 1).

The SADI plug-in to the IO Informatics Knowledge Explorer [24, 25] (KE) provides menu-driven access to the SHARE registry through a context menu that appears when right-clicking a piece of biological data on the KE canvas. In Figures 2A and B we show the same sample data from the examples above, loaded into the Knowledge Explorer. A right click reveals the “Find SADI Services” menu option, which then initiates a search based on the data-type that was selected. Here we have chosen the “approved-name” service from the resulting services menu by clicking the selection box. In Figure 2C the approved name for HGNC:7 has been added as new information to the canvas. Figure 2D shows the final result after a series of OpenLifeData2SADI services have been executed, following the workflow path in Figure 1.

### SHARE

The SHARE client [14] is one of several SADI clients capable of chaining multiple services together. We will utilize this client to emphasize the fact that, as a result of exposing OpenLifeData data as SADI services, it is no longer necessary to know which data exists in which of the 26+ OpenLifeData SPARQL endpoints. In this use case we imagine that a researcher has studied some human condition, has narrowed-down to a specific gene list of interest, and now wants to know more about those genes, their functions, and whether or not the proteins might be suitable drug targets based on known protein information from their respective Mouse homologues. Diagrammatically, the workflow is as shown in Figure 2 (using the service numbers and starting-data from above).

The SHARE interface is at http://dev.biordf.org/cardioSHARE. SHARE exposes SADI Web Services as if they were combined into a single, global, SPARQL endpoint. The SHARE SPARQL query that will invoke the workflow from Figure 2 is:


Note that it was not necessary to know which endpoint contained which data elements, nor to use “service” queries to federate over these endpoints. This is important when considering the complex structure of federated SPARQL queries, where it is necessary to know the location of the endpoint, and in some cases, the named-graph that must be queried. For example, the equivalent SPARQL query over the OpenLifeData endpoints, would be as follows:


As such, we believe that OpenLifeData2SADI makes the exploration across the more than 20 OpenLifeData data endpoints considerably more straightforward.

### Galaxy

**Figure 3:**
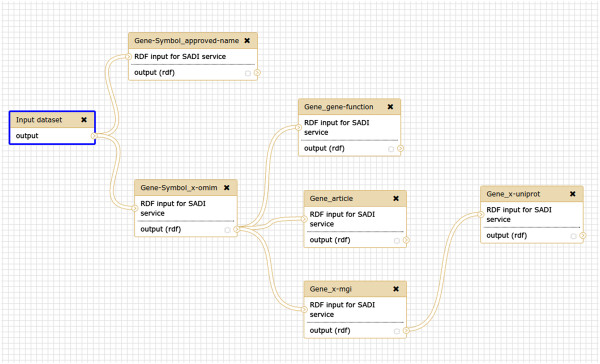
**A workflow of OpenLifeData2SADI services in the Galaxy workbench environment.** This workflow is an instantiation of the template workflow in Figure 1.

The Galaxy [26] workflow environment is very popular among life scientists, yet to date, we know of no Galaxy workflow that accesses OpenLifeData or Bio2RDF data. This is likely due to the lack of life science tools and services that deal with RDF-formatted data at all, and the lack of a straightforward template for mapping data between a workflow and a SPARQL query (and back again). The SADI Galaxy plugin [27, 28] provides SADI services as normal Galaxy tools [29], thus making it straightforward to chain OpenLifeData services together in the Galaxy environment. Figure 3 shows the same workflow as above, created within the Galaxy workbench. In order to reproduce the workflow, it is necessary to create, for yourself, a user on our Galaxy server [30] and import the history and workflow [31, 32]. The first item of the history can be used as the input to the workflow to reproduce the results reported here.

### Limitations and scalability

The use of SADI to expose data in SPARQL endpoints clearly adds a certain amount of overhead with respect to both execution-time and computational load; however, it is difficult to directly compare the two scenarios because (a) speed and load depend on the client, and web service clients are significantly different from one another, and from SPARQL clients; (b) the time (and knowledge) required to manually construct each desired SPARQL query, compared to the automated dynamic discovery of appropriate SADI services, is not considered in a head-to-head comparison of their respective execution times, and (c) It is considerably easier to optimize the execution plan for a SPARQL query, versus a service workflow. Nevertheless, a direct comparison of the “federated” query entered into the SHARE client (above) versus the equivalent federated SPARQL query entered into the Virtuoso web-based query interface, showed execution times of 34-39 seconds for SHARE compared to 2-3 seconds for Virtuoso. Thus, while the overhead of the Web Service solution is, in this case, significant, we feel it is still within reason for a user-interface, given the difficulty a user would face in creating and debugging SPARQL queries. Similarly, we would argue that the dynamically-generated, menu-driven interface provided by the KE plugin is orders of magnitude faster than the user having to manually type each SPARQL query into the KE SPARQL interface.

## Conclusions

In this work we attempted to address four distinct problems:

The first is that, since most bioinformatics workflows combine a variety of different kinds of Web Services together with local processors to execute the data retrieval and analysis, it is highly desirable to expose SPARQL endpoints in a discoverable manner akin to Web Services. Current approaches to exposing SPARQL endpoints as services result in services with low discoverability and incomplete (or even absent) descriptions of what will be returned from a service invocation. By exposing the contents of OpenLifeData as SADI Web Services, it becomes straightforward to integrate these endpoints into popular workflow environments such as Taverna [33] or Galaxy, and more importantly, the service interface, and the data that passes through the service, is explicitly semantically defined.

Second, the discovery of data in Bio2RDF (or any SPARQL endpoint) has, historically, required considerable prior knowledge and often trial-and-error exploration until an appropriate SPARQL query has been constructed. By enhancing the semantics of Bio2RDF, and indexing all of its semantically rich entity-relationships in the recent release of OpenLifeData, it became possible to expose all of this data as SADI services that are registered in the SHARE registry. It is now straightforward to discover, through highly predictable SPARQL queries, which Bio2RDF endpoints contain data of interest, and what the nature of that data is. Moreover, because the registry query is predictable, it is trivial to make a comprehensive map of all entity-to-entity connections within the entire OpenLifeData “universe”, even spanning between separate OpenLifeData endpoints, and this was made publicly available as an abstract workflow template following the Open Provenance Model for Workflows (OPMW) ontology [20, 21].

Third, SPARQL endpoints are a highly granular approach to bioinformatics data publishing akin to publicly exposing the SQL interface to a relational database. Historically, there have been very few core bioinformatics data hosts who allowed such fine-grained access to their databases, primarily because of the potential for users to submit resource-hungry queries (either accidentally, or on purpose). Indeed, this has already been identified as a problem with respect to queries against the UniProt SPARQL endpoint [34]. While losing the potential for query optimization, exposing RDF data via SADI Services has several advantages over exposing RDF triple-stores as SPARQL. First, because SADI services can be executed in a multiplexed manner, and asynchronously, bulk data requests could be easily managed over the available compute-resources at the host site. This is difficult to achieve with existing SPARQL endpoints. Second, because the Service exposes the RDF data via a ‘wrapper’ around a simple SPARQL query that is guaranteed to be correct, it becomes impossible for the data request to be malformed or resource-consuming (beyond what the provider allows). Thus, the data provider is better-shielded from misuse or abuse.

Finally, in these early days of Linked Data publishing in the life sciences, Linked Data resources can, justifiably, change as the data providers adapt their models, publishing procedures, publisher metadata and even endpoint locations and access restrictions to accommodate new behaviors or concerns. As such, SPARQL queries that are successful one day, may not be successful the next. By serving OpenLifeData through SADI Services, which are dynamically discovered by clients that automatically configure themselves for correct access, we provide an API that is much more resilient to underlying change than a pure SPARQL interface would be; updating the OpenLifeData2SADI behavior is simply a matter of changing the configuration file, or (in the worst case) updating one simple piece of code, compared to all users of the data being required to update their own software.

For all of these reasons, we feel that the availability of OpenLifeData2SADI will dramatically enhance the access to, and utility of, OpenLifeData for all biologists. This, in turn, will hopefully spur a more rapid adoption of both Linked Data and Semantic Web Services throughout the life science data provider community.

## Availability and requirements

The Java code and Perl scripts for this project are available on the Wilkinson laboratory Github, at https://github.com/wilkinsonlab/OpenLifeData2SADI under the Apache version 2 license. Indexing the OpenLifeData endpoints can be executed in either Java or Perl. Ontology and configuration file creation requires Java 6, Jena, and the OWL API. Serving the data as Web Services is accomplished by running the indexer (in Java or Perl), building the configuration files, and deploying the ‘SADI’ Perl script, with core dependencies on the SADI::Simple and RDF::Query::Client libraries from CPAN. The repository also contains the output files from the most recent execution of the OpenLifeData2SADI2OPMW.pl script, and these are free to use in any way.

## Authors’ information

MD is the lead investigator of the OpenLifeData project and is Associate Professor at Stanford University. MDW is the founder and leader of the SADI project, Issac Peral Distinguished Researcher at the Center for Plant Biotechnology and Genomics, and Director of the FBBVA-UPM Chair in Biological Informatics at the Universidad Politécnica de Madrid. JCT, AC, AG, MEA and ARG are, or have been, researchers in these collaborating laboratories during the execution of this project.

## Abbreviations

DBCLS: Database center for life science
HTTP: Hypertext transport protocol
NBDC: National bioscience database center
OPMW: Open provenance model for workflows
OWL: Web ontology language
RDF: Resource description framework
SPARQL: SPARQL Protocol and RDF Query Language
SADI: Semantic automated discovery and integration
SPO: Subject-predicate-object
VoID: Vocabulary of interlinked datasets.
